# Effects of caffeinated gum doses combined with post-activation performance enhancement on bench-press bar velocity: a randomized crossover study

**DOI:** 10.3389/fnut.2026.1828179

**Published:** 2026-05-26

**Authors:** Ziyu Wang, Bopeng Qiu, Penglin Diao, Yinkai Zhang, Jinxuan Bao, Zezhao Chen, Lehai Lin, Carl Petersen, Aleksandra Filip-Stachnik, Juan Del Coso

**Affiliations:** 1 Sports Coaching College, Beijing Sport University, Beijing, China; 2 Faculty of Health, University of Canterbury, Christchurch, New Zealand; 3 School of Strength and Conditioning, Beijing Sport University, Beijing, China; 4 Institute of Sport Sciences, Academy of Physical Education in Katowice, Katowice, Poland; 5 Sport Sciences Research Centre, Rey Juan Carlos University, Fuenlabrada, Spain

**Keywords:** boxing, exercise performance, intermittent sports, resistance exercise, sports nutrition

## Abstract

**Background:**

Evidence from randomized crossover trials indicates that both caffeine and post-activation performance enhancement (PAPE) can independently improve acute performance, but findings on their combined effects remain inconclusive. Therefore, this study examined whether 3 mg/kg or 6 mg/kg of caffeine via gum, when paired with a PAPE conditioning activity, would enhance bench-press bar velocity and total repetitions.

**Methods:**

Using a randomized, double-blind, placebo-controlled crossover design, 15 male amateur boxers completed six conditions: (a) placebo gum without PAPE (NoPAPE-0); (b)3 mg/kg of caffeine via gum without PAPE (NoPAPE-3); (c) 6 mg/kg of caffeine via gum without PAPE (NoPAPE-6); (d) PAPE + placebo gum (PAPE-0); (e) PAPE + 3 mg/kg of caffeine via gum (PAPE-3), and (f) PAPE + 6 mg/kg of caffeine via gum (PAPE-6). In PAPE conditions, participants performed one set of 3 repetitions of bench press exercise at 90% 1RM. Immediately afterward, participants chewed the assigned gum for 10 min, after which they began the performance test including four 12-s bench-press sets at 60% 1RM, performed at maximal intended velocity with 2-min rests. Mean and peak bar velocity and total repetitions were analyzed with linear mixed models treating with Condition coded as a 2 × 3 factorial (PAPE: No vs. Yes; Dose: 0, 3, 6 mg/kg of caffeine), including planned contrasts for dose effects within PAPE level and for PAPE vs. NoPAPE at matched doses.

**Results:**

Overall, PAPE produced significant higher mean velocity (+0.018 m/s) and peak velocity (+0.035 m/s) than NoPAPE across all caffeine doses (all *p* < 0.0001). Independently of PAPE, 3 mg/kg and 6 mg/kg of caffeine significantly improved velocity relative to placebo: mean velocity increased by +0.013–0.015 m/s for 3 mg/kg of caffeine and by +0.017–0.018 m/s for 6 mg/kg of caffeine with no differences between caffeine doses. For total repetitions, PAPE statistically exceeded NoPAPE only under placebo conditions (+2.87 reps; *p* < 0.0001). Caffeine significantly increased repetitions in NoPAPE (3 mg/kg and 6 mg/kg of caffeine > placebo; both *p* < 0.0001), but adding caffeine to PAPE did not increase repetition volume.

**Conclusion:**

Both PAPE and caffeinated gum significantly enhanced bench press bar velocity while combining PAPE with caffeinated gum yields greater performance benefits than caffeinated gum alone, irrespective of dose. In contrast, caffeine alone increased repetition volume, and PAPE did not augment this effect. Practically, athletes seeking to maximize bar velocity may combine PAPE with 3 mg/kg of caffeine via gum, whereas those aiming to increase repetition volume may rely on caffeine alone.

## 1 Introduction

Caffeine is widely used in both training and competition across various sports for its ergogenic effects (1, 2). It acts centrally by antagonizing adenosine receptors (3, 4), which enhances neurotransmission, lowers perceived exertion during exercise, and decreases muscle pain (5), and may also improve muscle contractility via calcium mobilization and Na^+^/K^+^ pump activation (6). Extensive research indicates that caffeine can enhance aerobic endurance, muscular strength and power, and a range of sport-specific performance outcomes (3, 7–9). All these ergogenic effects during exercise trials are most consistently observed with moderate doses of caffeine ranging from 3 to 6 mg/kg, typically ingested 30–60 min prior to exercise (10).

While most evidence supporting caffeine's ergogenic effects has been derived from studies using capsule, tablet and caffeinated drinks (10–12), alternative delivery forms such as caffeinated chewing gum have gained attention for their faster buccal absorption (13, 14) and potential advantages in time-sensitive, intermittent, or high-intensity athletic contexts (15, 16). This is because caffeinated gum produces a distinct pharmacokinetic profile compared with other delivery forms, such as capsules, or caffeinated drinks, enabling rapid buccal mucosa absorption (13, 17). This quick onset may be advantageous in time-sensitive or intermittent performance contexts (18).

Post-Activation Performance Enhancement (PAPE) is another acute performance-enhancement strategy, as it enhances subsequent performance via brief high-intensity conditioning activity (CA) performed a few minutes before the onset of the target performance activity (19). In upper-body bench-press protocols, performance benefits are often reported after longer recoveries (commonly ~8–12 min) (20). One study reported that performance improvements after a 3RM bench-press CA (3 reps) could still be observed at later sampling points (e.g., ~8–16 min) (21).

Concurrent use has produced amplified improvements in explosive actions such as vertical jumping, cycling sprints, and upper-body ballistic exercises, as well as modest gains in repeated high-intensity efforts (22, 23). Interestingly, these studies have administered caffeine exclusively in capsule or beverage form at doses of 3–6 mg/kg of caffeine (25, 27), leaving unclear whether alternative delivery forms, such as caffeinated gum, support similar combination strategies (23, 24). Because PAPE is transient and highly dependent on the recovery interval after the CA (19, 25, 26), the rapid absorption of caffeinated gum may allow caffeine administration closer to the intended performance window, thereby facilitating coincidence with the PAPE response. Administering caffeine immediately after CA and initiating the upper limb repeated burst task 10 min later represents a timing strategy limited direct investigation in PAPE × caffeine studies.

To the best of our knowledge, no studies have directly examined whether caffeinated gum modifies the combined response to PAPE during upper-body repeated-effort performance. Existing studies have used only a single caffeine dose, so it remains unclear whether 3 mg/kg and 6 mg/kg of caffeine interact differently with PAPE or whether either dose provides an advantage over PAPE alone. In addition, most of the available evidence focuses on lower-body tasks, with limited data on upper-body, multi-repetition performance. Thus, this study aimed to examine whether 3 mg/kg and 6 mg/kg of caffeine via gum, when combined with a PAPE protocol, would enhance bench-press bar velocity in a randomized, double-blind crossover design. Therefore, we hypothesized that caffeine administered via chewing gum would enhance bench-press bar velocity when combined with a PAPE protocol, with the 6 mg/kg dose expected to produce greater improvements than 3 mg/kg, and both caffeine conditions expected to outperform PAPE alone.

## 2 Method

### 2.1 Participants

Fifteen male apparently healthy amateur boxers (*n* = 15; see Table 1 for demographics) competing at the regional level were recruited. Inclusion criteria were: males aged 18–30 years; free from neuromuscular, cardiovascular, or metabolic disease; ≥2 years of systematic boxing and resistance-training experience; familiar with the bench-press exercise and 1RM testing. Exclusion criteria were: current musculoskeletal injury; known caffeine hypersensitivity or habitual caffeine intake of a moderate consumer level or higher (i.e., >3 mg/kg of caffeine or higher) (27); smoking; and habitual alcohol abuse. Although the primary analyses were conducted using linear mixed-effects models, an *a priori* sample size estimation was performed in G^*^Power 3.1 using an equivalent repeated-measures ANOVA design (within factors). The analysis assumed a small effect size (*f* = 0.18), an alpha level of 0.05, and statistical power of 0.80 (beta = 0.20), with 1 group, 6 measurements, a correlation of 0.90 among repeated measures (28). The assumed correlation and effect size were based on previous studies examining caffeine supplementation and muscular strength/power outcomes in resistance-trained men. The estimated minimum sample size was 8 participants. To account for potential drop-out, 15 amateur boxers were recruited. During data collection, participants abstained from any form of dietary caffeine for 24 h, and strenuous exercise for 24 h before each trial; diet was replicated using a 24-h food recall, and sessions were scheduled between 08:00 and 12:00 at the same time of day (±1 h) to minimize circadian effects. This study was conducted in accordance with the ethical standards of the Declaration of Helsinki. The study protocol was reviewed and approved by the institutional ethics committee (Approval No. 2024496H).

**Table 1:** Baseline demographics.

Measurement	Mean ±*SD*
Age (year)	19 ± 1
Height (cm)	174.2 ± 5.1
Body mass (kg)	75.3 ± 8.1
Boxing experience (years)	4.2 ± 1.8
Bench press 1RM (kg)	97.37 ± 27.35

### 2.2 Pre-experimental standardization

Participants maintained usual hydration and dietary habits (including pre-workout meals) and habitual caffeine intake throughout the study (23), registering food intake 24 h prior to the first trial (18). To standardize within-subject diet, they replicated the same dietary pattern before subsequent trials. Habitual caffeine intake was measured via a modified Bühler et al. (29) validated questionnaire, assessing intake 4 weeks pre-experiment as recommended (27). Habitual caffeine intake was 68 ± 23 mg/day, corresponding to < 3 mg/kg day in all participants, and no participant exceeded 200 mg/day.

### 2.3 Experimental design

A randomized, double-blind, placebo-counterbalanced crossover design was employed, with each participant serving as his own control. Participants first underwent two preliminary sessions to record anthropometric data and determine 1-RM bench press strength on a Smith machine, guided by certified strength coaches following standard NSCA procedures (30). Then, participants completed six experimental conditions separated by 72-h intervals, following a 2 (PAPE: No vs. Yes) × 3 (Dose: 0 vs. 3 vs. 6 mg/kg of caffeine) factorial structure: (a) placebo gum without PAPE (NoPAPE-0) (b) 3 mg/kg of caffeine via gum without PAPE (NoPAPE-3); (c) 6 mg/kg of caffeine via gum without PAPE (NoPAPE-6); (d) PAPE + placebo gum (PAPE-0); (e) PAPE + 3 mg/kg of caffeine via gum (PAPE-3), and (f) PAPE + 6 mg/kg of caffeine via gum (PAPE-6). During each 72-h interval between experimental conditions, participants were instructed to maintain similar habitual training and daily physical activity patterns across all trials.

Military Energy Gum (MEG^®^, USA; 100 mg caffeine per piece) was used to provide the caffeine doses. Partial pieces were weighed to ensure that the final caffeine content matched the prescribed dose. Placebo gums were identical in appearance and flavor to maintain blinding. Both caffeine and placebo gums were dispensed by the investigator from identical sealed containers directly into the participants' mouths, so that participants could not see the gums or any packaging, thereby preserving blinding.

For all conditions, participants completed a general warm-up consisting of 5 min of low-intensity cycling followed by 5 min of dynamic upper-body stretching exercises (31). The conditioning activity consisted of the bench press exercise performed on a Smith machine. For the NoPAPE conditions, the specific warm-up consisted of one set of 8 repetitions at 30% of 1-RM and one set of 5 repetitions at 45% of 1-RM, with 3-min rest intervals between sets. For the PAPE conditions (PAPE-0, PAPE-3, PAPE-6), the specific warm-up consisted of 8 repetitions at 30% of 1-RM and 5 repetitions at 45% of 1-RM, followed by a conditioning activity of 3 repetitions at 90% of 1-RM with 3-min rest intervals between sets (31).

As soon as the warm-up was completed in the NoPAPE trials, or the conditioning activity was finished in the PAPE trials, participants started chewing the assigned gum (placebo or caffeine) for 10 min while seated. After this 10-min period, they discarded the gum and began the bench-press protocol within 30 s. Thus, the time elapsed between the end of the warm-up/conditioning activity and the onset of the first test set was approximately 10 min in all conditions (32, 33). This fixed interval was chosen to standardize timing across conditions and to target a recovery window commonly sampled in bench-press CA studies, where performance benefits are frequently observed at ~8–12 min and has also been observed at later sampling points (21). The four-set performance assessment was completed within approximately 16 min after the end of CA. This timing approach is consistent with bench-press PAPE study designs that assess performance after a standardized recovery interval following the CA (31).

All warm-up, conditioning activity and test sets were performed as a flat bench-press exercise on a Smith machine. Participants lay in a supine position with their feet flat on the floor, the head, shoulders and buttocks in contact with the bench, and a pronated grip slightly wider than shoulder-width. The bar was lowered in a controlled manner to lightly touch the chest and then pressed upward until the elbows were almost fully extended, without bouncing or lifting the hips off the bench.

Following each experimental condition, participants completed a bench-press performance test on a Smith machine. The test consisted of four 12-s sets at 60% of 1-RM, separated by 2-min passive recovery intervals. At the beginning of each set, an audio signal was given and participants were instructed to perform as many repetitions as possible, using maximal intended barbell velocity during the concentric phase while controlling, but not deliberately slowing, the eccentric phase. Although eccentric tempo was not externally paced, participants were instructed to keep the eccentric phase consistent across repetitions and conditions and to avoid bouncing; therefore, bar-velocity outcomes were prioritized as the main performance measure, whereas total repetitions were interpreted as an additional outcome.

**Figure 1:**
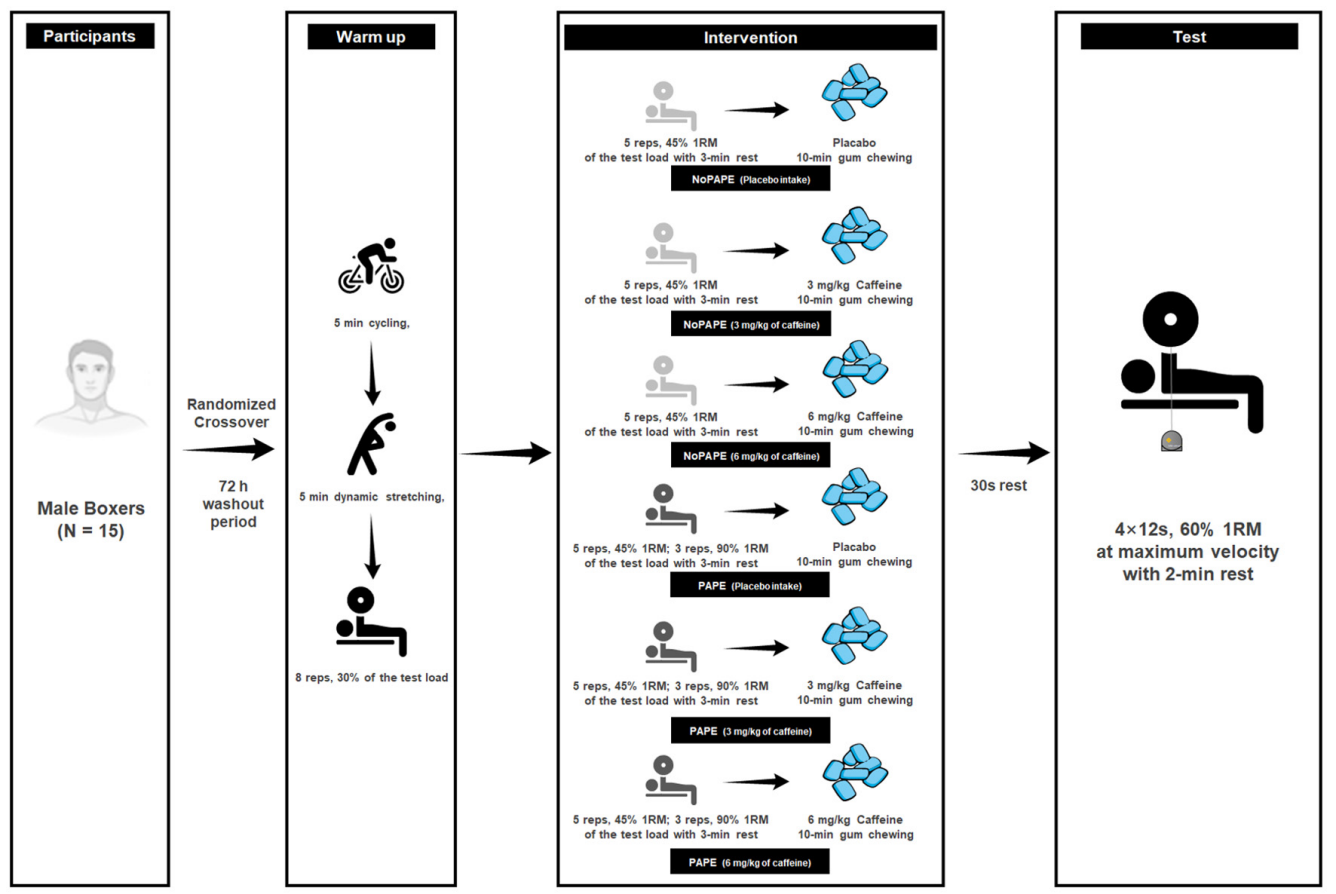
Overview of the randomized crossover protocol, showing the six experimental trials (NoPAPE-0, NoPAPE-3, NoPAPE-6, PAPE-0, PAPE-3, PAPE-6), the warm-up procedures with or without the PAPE conditioning activity, the 10-min gum-chewing period (placebo or caffeine), and the subsequent bench-press performance test.

Two researchers stood on either side of the bar to monitor technique, ensure a consistent range of motion, and provide standardized verbal encouragement. The load of 60% 1-RM was chosen because it combines the relatively high mean surface EMG activity observed with heavier loads with the high total integrated EMG (31, 34) (Figure 1).

### 2.4 Measurements

Bar velocity output was recorded using a linear position sensor (GymAware; Kinetic Performance Technologies, Canberra, Australia), with the sensor string perpendicular to the Smith machine barbell. A small weight was hung from the barbell to the floor before each condition to set sensor position and ensure accurate vertical velocity measurement. The smallest possible weight increment on the Smith machine was 2.5 kg, which was used to ensure consistent and repeatable loading. The validity and reliability of this system have been described elsewhere (35). The average barbell velocity was determined as the average of all the average velocities of the repetitions in each set. Peak barbell velocity was averaged over all repetitions of all peaks in each set. The ICCs for these measurements in our laboratory were as follows: mean velocity [0.957 (95% *CI*: 0.936–0.978)], and peak velocity [0.964 (95% *CI*: 0.944–0.981)]. Height and body mass were measured using an InBody bioelectrical impedance analyzer (InBody Co., Ltd., Seoul, Korea), with values recorded to the nearest 0.1 cm and 0.1 kg, respectively.

### 2.5 Statistical analyses

Analyses were performed in R 4.3.0 (R Foundation for Statistical Computing). The within-participant condition factor was coded as a 2 × 3 factorial with PAPE (NoPAPE vs. PAPE) and caffeine dose (placebo [0 mg/kg of caffeine], 3 mg/kg of caffeine, and 6 mg/kg of caffeine). The main performance outcomes were mean and peak barbell velocity for each set and condition. As an additional performance outcome, we also analyzed the total number of repetitions completed per condition, calculated as the sum of repetitions across the four sets within each PAPE × dose combination.

For all outcomes, we fitted linear mixed-effects models estimated by restricted maximum likelihood. PAPE, dose and their interaction were specified as fixed effects. To account for the repeated-measures structure, we included random intercepts for each participant-by-condition combination (id: condition) and random slopes for Set at the participant level for the set-wise velocity outcomes; for total repetitions (aggregated across sets) only random intercepts for participants were included. For tests of linear dose–response trends, dose was additionally treated as an ordinal numeric variable coded 0, 1, and 2 for placebo, 3 mg/kg of caffeine and 6 mg/kg of caffeine, respectively.

## 3 Result

### 3.1 Mean velocity

**Figure 2:**
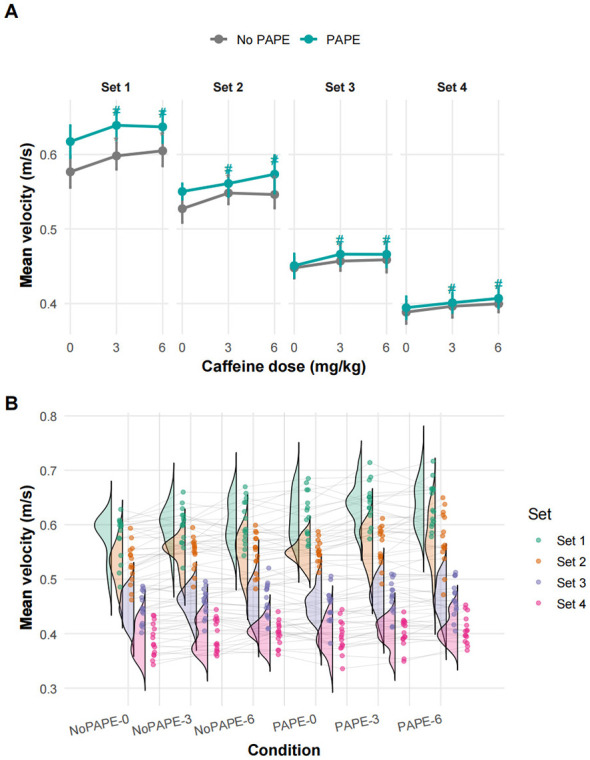
Mean bar-velocity responses to caffeine with and without PAPE. **(A)** Estimated marginal means of mean bar velocity (±95% confidence intervals) for each set (Set 1–Set 4) as a function of caffeine dose (0, 3, 6 mg/kg) and PAPE condition (No PAPE vs. PAPE). Symbols above the points indicate doses that are significantly higher than 0 mg/kg within the corresponding PAPE condition (adjusted *p* < 0.05). * and # indicate doses that are significantly higher than 0 mg/kg in the No PAPE and PAPE conditions, respectively (adjusted *p* < 0.05). **(B)** Raincloud plot showing the distribution of individual mean bar-velocity values across the six experimental trials (NoPAPE-0, NoPAPE-3, NoPAPE-6, PAPE-0, PAPE-3, PAPE-6).

Aggregating the four sets (Table 2), PAPE significantly outperformed NoPAPE (+0.0179, 95% *CI* 0.0144–0.0214, *p* < 0.0001). At each dose (0/3/6), simple effects favored PAPE [0 mg/kg of caffeine (i.e., placebo) + 0.0182, 3 mg/kg of caffeine + 0.0169, 6 mg/kg of caffeine + 0.0185; all *p* < 0.0001], indicating a consistent benefit across doses. Within-condition contrasts showed that, in the PAPE condition, 3 mg/kg of caffeine and 6 mg/kg of caffeine exceeded 0 mg/kg of caffeine (+0.0136 and +0.0177, respectively; both *p* < 0.0001) without differences between 3 vs. 6 mg/kg of caffeine (+0.0040, *p* = 0.534). The NoPAPE condition followed the same pattern (3 mg/kg of caffeine > 0 mg/kg of caffeine and 6 mg/kg of caffeine > 0 mg/kg of caffeine: +0.0149 and +0.0173, respectively; both *p* < 0.0001; without differences between 3 vs. 6 mg/kg of caffeine: +0.0025, *p* = 0.629. Joint tests detected no dose-by-set interaction, supporting presentation aggregated across the four sets. Dose–response curves and distributions across the six conditions are shown in Figure 2.

**Table 2:** Mean velocity results across six experimental conditions (NoPAPE-0, NoPAPE-3, NoPAPE-6, PAPE-0, PAPE-3, PAPE-6) during four 12-s bench-press sets, with planned comparisons between PAPE and NoPAPE at matched caffeine doses and within-condition dose effects of 0, 3, and 6 mg/kg of caffeine.

Section	Contrast	Estimate	95% *CI* lower	95% *CI* upper	*p*
A. PAPE vs. NoPAPE	PAPE (0 mg/kg of caffeine) – NoPAPE (0 mg/kg of caffeine)	0.0182	0.0125	0.0238	< 0.0001
A. PAPE vs. NoPAPE	PAPE (3 mg/kg of caffeine) – NoPAPE (3 mg/kg of caffeine)	0.0169	0.0096	0.0242	< 0.0001
A. PAPE vs. NoPAPE	PAPE (6 mg/kg of caffeine) – NoPAPE (6 mg/kg of caffeine)	0.0185	0.0139	0.0230	< 0.0001
B. PAPE dose pairwise comparisons	PAPE (3 mg/kg of caffeine) – PAPE (0 mg/kg of caffeine)	0.0136	0.0084	0.0187	< 0.0001
B. PAPE dose pairwise comparisons	PAPE (6 mg/kg of caffeine) – PAPE (0 mg/kg of caffeine)	0.0176	0.0106	0.0246	< 0.0001
B. PAPE dose pairwise comparisons	PAPE (6 mg/kg of caffeine) – PAPE (3 mg/kg of caffeine)	0.0040	−0.0049	0.0129	0.5336
C. NoPAPE dose pairwise comparisons	NoPAPE (3 mg/kg of caffeine) – NoPAPE (0 mg/kg of caffeine)	0.0149	0.0072	0.0224	< 0.0001
C. NoPAPE dose pairwise comparisons	NoPAPE (6 mg/kg of caffeine) – NoPAPE (0 mg/kg of caffeine)	0.0173	0.0106	0.0239	< 0.0001
C. NoPAPE dose pairwise comparisons	NoPAPE (6 mg/kg of caffeine) – NoPAPE (3 mg/kg of caffeine)	0.0024	−0.0039	0.0088	0.6293

Linear mixed model with Satterthwaite df; four sets combined. Part A: PAPE vs. NoPAPE at the same dose (preplanned, unadjusted). Parts B/C: within-group dose comparisons (Tukey-adjusted). Dose × set interactions were non-significant in joint tests.

### 3.2 Peak velocity

**Figure 3:**
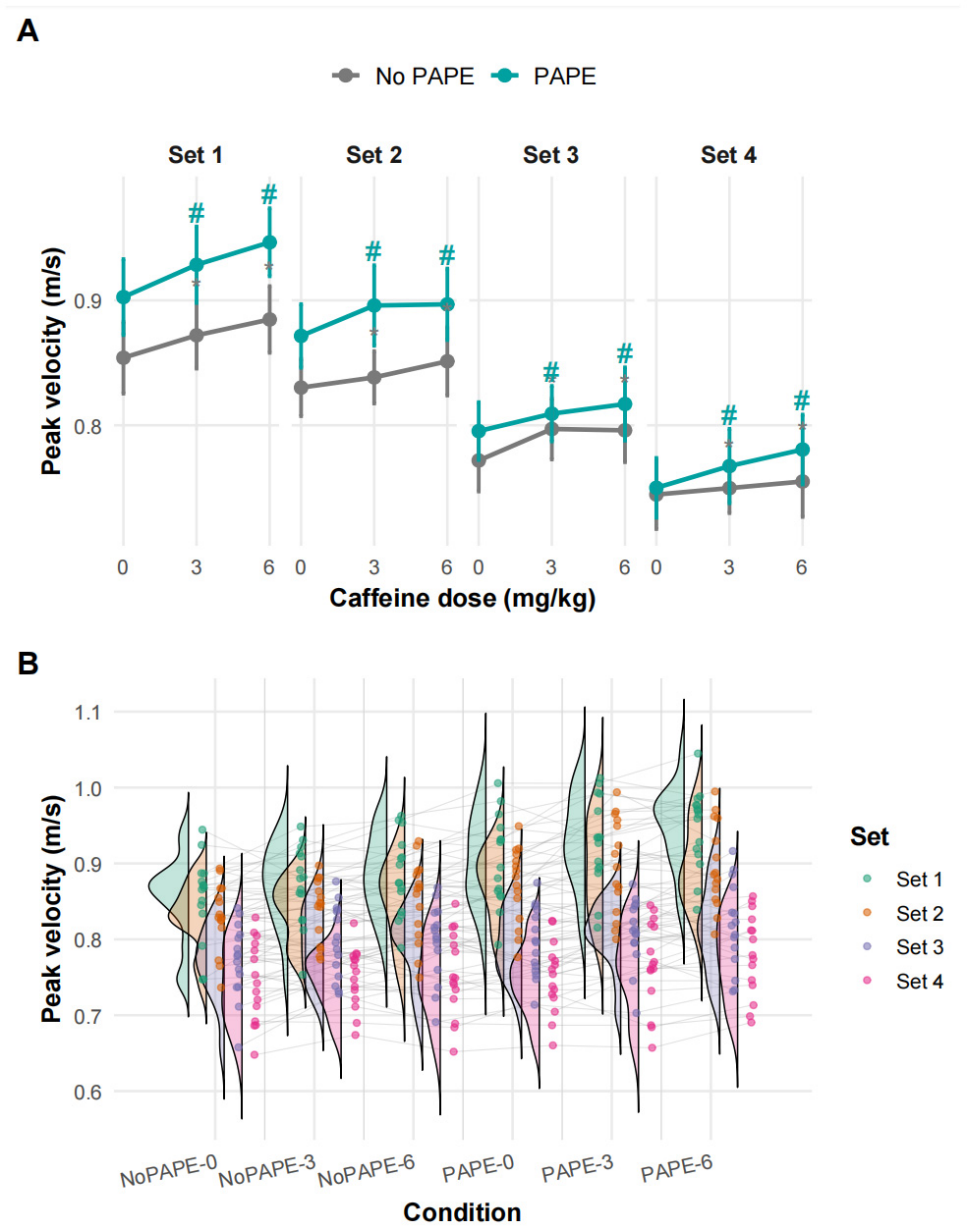
Peak bar-velocity responses to caffeine with and without PAPE. **(A)** Estimated marginal means of peak bar velocity (±95% confidence intervals) for each set (Set 1–Set 4) as a function of caffeine dose (0, 3, 6 mg/kg) and PAPE condition (No PAPE vs. PAPE). Symbols above the points indicate doses that are significantly higher than 0 mg/kg within the corresponding PAPE condition (adjusted *p* < 0.05). * and # indicate doses that are significantly higher than 0 mg/kg in the No PAPE and PAPE conditions, respectively (adjusted *p* < 0.05). **(B)** Raincloud plot showing the distribution of individual peak bar-velocity values across the six experimental trials (NoPAPE-0, NoPAPE-3, NoPAPE-6, PAPE-0, PAPE-3, PAPE-6).

Aggregating the four sets (Table 3), PAPE significantly outperformed NoPAPE (+0.0347, 95% *CI* 0.0294–0.0401, *p* < 0.0001). At each caffeine dose, effects favored PAPE over NoPAPE (0 mg/kg of caffeine +0.0297 [0.0226–0.0369], 3 mg/kg of caffeine +0.0360 [0.0260–0.0460], 6 mg/kg of caffeine +0.0385 [0.0305–0.0465]; all *p* < 0.0001), indicating a consistent benefit of PAPE across doses. The within-condition dose contrasts showed that, in the PAPE condition, 3 mg/kg of caffeine > 0 mg/kg of caffeine (+0.0204, *p* = 0.0002) and 6 mg/kg of caffeine > 0 mg/kg of caffeine (+0.0304, *p* < 0.0001), whereas the comparison 6 mg/kg of caffeine vs. 3 mg/kg of caffeine did not reach statistical significance (+0.0100, *p* = 0.0827). The within-condition dose contrasts in the NoPAPE condition followed the same pattern (3 mg/kg of caffeine > 0 mg/kg of caffeine: +0.0141, *p* = 0.0006; 6 mg/kg of caffeine > 0 mg/kg of caffeine: +0.0216, *p* < 0.0001; 6 mg/kg of caffeine vs. 3 mg/kg of caffeine: +0.0075, *p* = 0.1846). Joint tests detected no dose-by-set interaction, supporting presentation aggregated across the four sets. Dose–response curves and distributions across the six conditions are shown in Figure 3.

**Table 3:** Peak velocity results across six experimental conditions (NoPAPE-0, NoPAPE-3, NoPAPE-6, PAPE-0, PAPE-3, PAPE-6) during four 12-s bench-press sets, with planned comparisons between PAPE and NoPAPE at matched caffeine doses and within-condition dose effects of 0, 3, and 6 mg/kg of caffeine.

Section	Contrast	Estimate	95% *CI* lower	95% *CI* upper	*p*
A. PAPE vs. NoPAPE	PAPE (0 mg/kg of caffeine) – NoPAPE (0 mg/kg of caffeine)	0.0297	0.0226	0.0369	< 0.0001
A. PAPE vs. NoPAPE	PAPE (3 mg/kg of caffeine) – NoPAPE (3 mg/kg of caffeine)	0.0360	0.0260	0.0460	< 0.0001
A. PAPE vs. NoPAPE	PAPE (6 mg/kg of caffeine) – NoPAPE (6 mg/kg of caffeine)	0.0385	0.0305	0.0465	< 0.0001
B. PAPE dose pairwise comparisons	PAPE (3 mg/kg of caffeine) – PAPE (0 mg/kg of caffeine)	0.0204	0.0087	0.0321	0.0002
B. PAPE dose pairwise comparisons	PAPE (6 mg/kg of caffeine) – PAPE (0 mg/kg of caffeine)	0.0304	0.0226	0.0382	< 0.0001
B. PAPE dose pairwise comparisons	PAPE (6 mg/kg of caffeine) – PAPE (3 mg/kg of caffeine)	0.0100	−0.0010	0.0210	0.0827
C. NoPAPE dose pairwise comparisons	NoPAPE (3 mg/kg of caffeine) – NoPAPE (0 mg/kg of caffeine)	0.0141	0.0055	0.0228	0.0006
C. NoPAPE dose pairwise comparisons	NoPAPE (6 mg/kg of caffeine) – NoPAPE (0 mg/kg of caffeine)	0.0216	0.0134	0.0298	< 0.0001
C. NoPAPE dose pairwise comparisons	NoPAPE (6 mg/kg of caffeine) – NoPAPE (3 mg/kg of caffeine)	0.0075	−0.0026	0.0175	0.1846

Linear mixed model with Satterthwaite df; four sets combined. Part A: PAPE vs. NoPAPE at the same dose (preplanned, unadjusted). Parts B/C: within-group dose comparisons (Tukey-adjusted). Dose × set interactions were non-significant in joint tests.

### 3.3 Total repetitions

**Figure 4:**
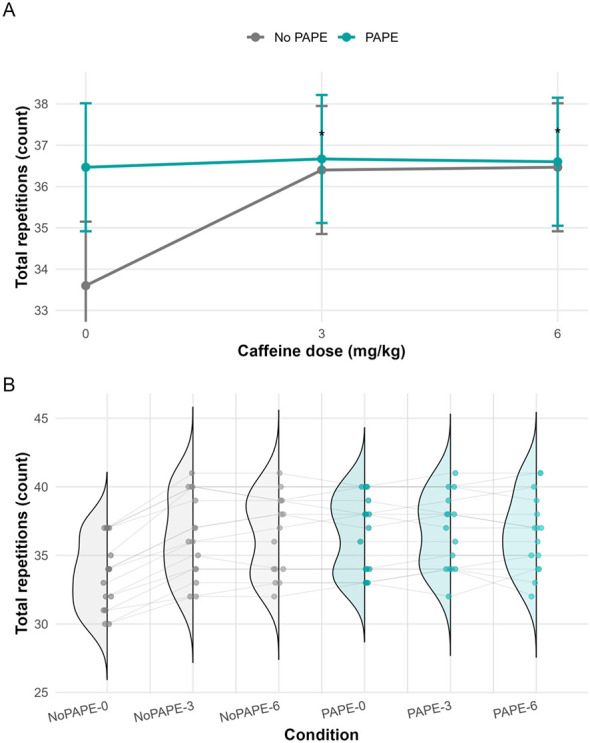
Total repetitions across the six experimental trials. **(A)** Estimated marginal means (±95% *CI*) for total repetitions as a function of caffeine dose (0, 3, 6 mg/kg) under NoPAPE and PAPE conditions, averaged across sets. Asterisks indicate significantly higher values than the corresponding NoPAPE-0 condition within the NoPAPE trials (Tukey-adjusted *p* < 0.05). **(B)** Distribution of individual total repetitions for each condition (NoPAPE-0, NoPAPE-3, NoPAPE-6, PAPE-0, PAPE-3, PAPE-6).

Across the three matched caffeine doses (Table 4), PAPE exceeded NoPAPE only in the 0 mg/kg of caffeine condition (+2.87 reps, 95% *CI* 2.41–3.32, *p* < 0.0001), whereas differences at 3 mg/kg of caffeine and 6 mg/kg of caffeine were small and non-significant (3 mg/kg of caffeine: +0.27 reps, 95% *CI* −0.19 to 0.72, *p* = 0.25; 6 mg/kg of caffeine: +0.13 reps, 95% *CI* −0.32 to 0.59, *p* = 0.56). Within the PAPE condition, there was no clear dose–response pattern (3 vs. 0 mg/kg of caffeine: +0.20 reps, *p* = 0.66; 6 vs. 0 mg/kg of caffeine: +0.13 reps, *p* = 0.83; 6 vs. 3 mg/kg of caffeine: −0.07 reps, *p* = 0.95). In contrast, within the NoPAPE condition, both 3 mg/kg of caffeine and 6 mg/kg of caffeine increased total repetitions relative to 0 mg/kg of caffeine (3 vs. 0 mg/kg of caffeine: +2.80 reps, 95% *CI* 2.25–3.35; 6 vs. 0 mg/kg of caffeine: +2.87 reps, 95% *CI* 2.32–3.42; both *p* < 0.0001), while the comparison between 6 mg/kg of caffeine and 3 mg/kg of caffeine remained non-significant (+0.07 reps, *p* = 0.95). Joint tests indicated significant main effects of PAPE and dose as well as a PAPE × dose interaction. Dose–response patterns for each condition are summarized in Figure 4.

**Table 4:** Total repetitions completed across six experimental conditions (NoPAPE-0, NoPAPE-3, NoPAPE-6, PAPE-0, PAPE-3, PAPE-6) during four 12-s bench-press sets, with planned comparisons between PAPE and NoPAPE at matched caffeine doses and within-condition dose effects of 0, 3, and 6 mg/kg of caffeine.

Section	Contrast	Estimate	95% *CI* lower	95% *CI* upper	p
A. PAPE vs. NoPAPE	PAPE (0 mg/kg of caffeine) – NoPAPE (0 mg/kg of caffeine)	2.8667	2.4099	3.3234	< 0.0001
A. PAPE vs. NoPAPE	PAPE (3 mg/kg of caffeine) – NoPAPE (3 mg/kg of caffeine)	0.2667	−0.1901	0.7234	0.2482
A. PAPE vs. NoPAPE	PAPE (6 mg/kg of caffeine) – NoPAPE (6 mg/kg of caffeine)	0.1333	−0.3234	0.5901	0.5623
B. PAPE dose pairwise comparisons	PAPE (3 mg/kg of caffeine) – PAPE (0 mg/kg of caffeine)	0.2	−0.7484	0.3484	0.6588
B. PAPE dose pairwise comparisons	PAPE (6 mg/kg of caffeine) – PAPE (0 mg/kg of caffeine)	0.1333	−0.6817	0.415	0.8301
B. PAPE dose pairwise comparisons	PAPE (6 mg/kg of caffeine) – PAPE (3 mg/kg of caffeine)	0.0667	−0.4817	0.615	0.9544
C. NoPAPE dose pairwise comparisons	NoPAPE (3 mg/kg of caffeine) – NoPAPE (0 mg/kg of caffeine)	2.8	2.2516	3.3484	< 0.0001
C. NoPAPE dose pairwise comparisons	NoPAPE (6 mg/kg of caffeine) – NoPAPE (0 mg/kg of caffeine)	2.8667	2.3183	3.415	< 0.0001
C. NoPAPE dose pairwise comparisons	NoPAPE (6 mg/kg of caffeine) – NoPAPE (3 mg/kg of caffeine)	0.0667	−0.615	0.4817	0.9544

Linear mixed model with Satterthwaite df; A: preplanned, unadjusted contrasts; B/C: Tukey-adjusted within-condition dose comparisons.

## 4 Discussion

The purpose of this study was to determine whether caffeinated chewing gum at doses of 3 mg/kg of caffeine or 6 mg/kg of caffeine, alone or combined with a single-set PAPE protocol, could enhance bench-press performance in amateur boxers. The results showed that both PAPE and caffeinated gum independently significantly improved mean and peak bar velocity, with small but consistent performance gains across conditions. Combining caffeinated gum with PAPE produced additional benefits for bar velocity, although the magnitude of improvement was modest and did not differ meaningfully between 3 mg/kg and 6 mg/kg of caffeine doses. In contrast, caffeinated gum alone increased total repetitions, whereas adding PAPE did not yield further volume improvements once caffeine was ingested.

Consistent with the planned contrasts, a PAPE produced small significant but robust gains in bar-velocity at every caffeine dose: across sets, PAPE exceeded NoPAPE for both mean velocity and peak velocity. Although the dose–response was shallow (3 and 6 mg/kg of caffeine outperforming placebo with no clear difference between 3 and 6 mg/kg of caffeine), at both active doses PAPE clearly exceeded NoPAPE for both mean and peak barbell velocity. This suggests that combining PAPE with caffeinated gum yields greater performance benefits than caffeinated gum alone, irrespective of dose. Total repetitions told a different story: PAPE surpassed NoPAPE only at 0 mg/kg of caffeine, with no advantage at 3 or 6 mg/kg of caffeine, whereas caffeinated gum increased repetitions within NoPAPE, yielding a significant PAPE × dose interaction for this outcome.

This study observed significant improvements in both peak velocity, mean velocity and total repetitions during bench press following caffeine gum intake, aligning with a recent meta-analysis (36), that reported caffeinated gum enhances strength and power exercise performance. Caffeinated gum demonstrated an overall effect comparable to caffeine capsules (36, 37). This aligns with similar plasma caffeine responses between gum and capsules, despite faster absorption with gum. Caffeine likely enhances bench press performance via central and peripheral mechanisms, including muscle contraction pathways (38). In our data, both 3 and 6 mg/kg of caffeine via gum improved bar velocity vs. 0 mg/kg of caffeine in both PAPE and NoPAPE, but 6 vs. 3 mg/kg of caffeine was not significant for mean velocity or peak velocity. No greater benefit was found with higher caffeine doses, as 3 mg/kg and 6 mg/kg of caffeine showed no significant differences in any performance parameters. For repetition count specifically, caffeinated gum increased repetitions only in NoPAPE (3, 6 > 0), and PAPE exceeded NoPAPE only at 0 mg/kg of caffeine, consistent with a PAPE × dose interaction for this outcome. Louise Jones et al. found both 3 mg/kg and 6 mg/kg of caffeine enhanced female lower-body muscular endurance without significant differences, though a slight effect size favored 3 mg/kg of caffeine (39). Consistent with these findings, our study observed higher caffeine doses might increase mean velocity and peak velocity, yet no significant improvements occurred at 6 mg/kg of caffeine compared to 3 mg/kg of caffeine.

Current data show near-maximal intensity (90% 1RM) bench press conditioning activity enhances subsequent bench press performance, with the most consistent advantages observed on bar-velocity outcomes. The observed gains are parsimoniously explained by transient potentiation mechanisms that increase force-generating capacity and bar speed (40). This finding aligns with prior research, highlighting benefits of low-volume high-intensity conditioning on subsequent performance in various sports (41).

Additionally, compared with the conditioning activity alone (PAPE), adding caffeinated gum improved subsequent bench-press velocity across sets, with statistically gains when contrasting PAPE-3 vs. PAPE-0 and PAPE-6 vs. PAPE-0. However, the magnitude of improvement was modest and not clearly dose-dependent: direct 6 vs. 3 mg/kg of caffeine contrasts were not significant for mean velocity or peak velocity, and joint tests indicated no dose × set interaction for velocity outcomes. Taken together, caffeinated gum appears to provide an additive benefit when combined with PAPE rather than a clear synergistic effect, and we found no consistent superiority of 6 over 3 mg/kg of caffeine for velocity measures. This pattern was outcome-specific: for total repetitions, adding caffeinated gum to PAPE did not increase volume, whereas in NoPAPE, both 3 mg/kg and 6 mg/kg of caffeine exceeded 0 mg/kg of caffeine. Moreover, PAPE surpassed NoPAPE only at 0 mg/kg of caffeine. Thus, while caffeinated gum + PAPE meaningfully increases bar velocity (a performance-relevant quality), it does not necessarily increase repetition count. Mechanistically, caffeine is likely related to increased activation of type II muscle fibers and phosphorylation of myosin regulatory light chains induced by conditioning activities (42), as well as caffeine's physiological effects on calcium ion mobilization (6), enhanced sodium/potassium pump activity, and adenosine receptor antagonism (5, 43).

Given the high correlation between bench press and punching in boxing (44), the results presented in this study are relevant to boxers and athletes who participate in sports characterized by high power output and speed (45). However, several limitations of the current study should be discussed to understand the scope of the findings. First, the sample consisted exclusively of young male amateur boxers; therefore, the results cannot be generalized to female athletes or to populations with different training backgrounds, levels of expertise, or age ranges. In addition, the relatively small sample size may have limited the ability to detect smaller effects or more complex interactions. Second, although bench-press performance may be associated with punching-related physical capacities, the study did not include boxing-specific performance assessments. Consequently, it remains unclear to what extent the observed improvements in bar velocity and repetition capacity translate to competitive boxing actions, such as punching frequency, punching force, or combinations performed under fatigue, and the present findings should therefore be interpreted primarily as evidence of changes in upper-body neuromuscular performance rather than direct improvements in boxing-specific performance. Third, the responses to both PAPE and caffeine may differ in lower-body or whole-body movements, and the present protocol focused only on an upper-body resistance task; thus, the transferability of these findings to movements with larger muscle mass involvement is uncertain. Fourth, side effects of caffeine were not assessed. This is an important limitation because any potential ergogenic benefit should be interpreted with caution in the absence of side-effect data, particularly at higher doses. Habitual caffeine intake was assessed prior to the experiment, and only participants with relatively low habitual caffeine intake were included. However, genetic factors that may influence caffeine responsiveness, such as CYP1A2 polymorphisms, were not assessed. Additionally, although a fixed 10-min recovery period was used to align with typical PAPE time courses and gum-based caffeine absorption kinetics, the optimal recovery duration may vary across individuals and caffeine doses; adjusting this interval might have altered the combined PAPE–caffeine response. Future studies should compare different recovery intervals to determine the optimal timing of the combined response. Collectively, these factors should be considered when applying the present findings to training or competition settings.

## 5 Conclusions

In bench-press exercise performed at maximal intended velocity, a single PAPE protocol consisting of one set of 3 repetitions at 90% 1RM produced small statistically significant but consistent improvements in both mean and peak bar velocity compared with a standard warm-up. Independently of PAPE, caffeinated gum at both 3 and 6 mg/kg enhanced bar-velocity outcomes relative to placebo, although no meaningful differences were observed between the two caffeine doses. Caffeinated gum also increased total repetition volume across sets, demonstrating a clear ergogenic effect on strength-endurance. When PAPE and caffeinated gum were combined, bar-velocity gains were additive but not synergistic, with caffeinated gum providing further improvement beyond PAPE alone. When repetition volume is prioritized, caffeine via gum (3–6 mg/kg) seems sufficient and PAPE may be optional once caffeine is used.

## Data Availability

The original contributions presented in the study are included in the article/supplementary material, further inquiries can be directed to the corresponding author.
